# Supplementary material for: Are carbon emissions trading and green financial instruments synergistic? -Comprehensive quantitative research based on content analysis
Source: PLoS One. 2024 Mar 7;19(3):e0298601. doi: 10.1371/journal.pone.0298601 (PMC10919601; doi:10.1371/journal.pone.0298601)
Supplement: S1 Data — (ZIP) [file pone.0298601.s001.zip › Data/地级市Z_F工作报告.zip!/╦─┤¿/╣π░▓╩╨/└┤╫╘╣1⁄2╓┌║┼í╛┬φ┐╦╩2╛▌═°í┐_2017.docx]

各位代表:

现在,我代表四届市人民政府,向大会报告过去五年的政府工作,并对今后五年和今年工作提出建议,请各位代表审查,并请市政协委员提出意见。

过去五年工作回顾

2012年以来的五年,是我市改革发展进程中极不平凡的五年。面对复杂多变的宏观经济形势和艰巨繁重的改革发展稳定任务,市政府在省委、省政府和市委的坚强领导下,在市人大、市政协的监督支持下,全面贯彻落实党的十八大、十八届三中、四中、五中、六中全会和习近平总书记系列重要讲话精神,牢固树立“五大发展理念”,紧紧围绕“两个跨越”目标,全力实施“三大发展战略”,发扬“逢山开路、遇水架桥、披荆斩棘、勇往直前”的广安精神,统筹推进稳增长、促改革、调结构、惠民生、防风险各项工作,全面完成“十二五”规划和市第四届人民代表大会第一次会议确定的各项目标任务,全市改革开放和现代化建设取得新的重大成就。

五年来,我们充分发挥伟人故里独特优势,抢抓机遇主动作为,广安战略位势和对外影响力大幅提升。成功争取并实施了一系列重大政策。川渝合作示范区、国家西部承接产业转移示范区等国家战略全面实施;国家中小城市综合改革试点城市、首批国家循环经济示范城市、国家现代农业示范区、首批国家商标战略实施示范城市、国家住宅产业现代化综合试点城市、国家智慧城市试点城市、部省共建教育改革发展试验区、嘉陵江流域国家生态文明先行示范区等国家政策在广安试点示范,为西部地区推进区域合作、承接产业转移、实现转型发展提供了鲜活经验。先后获得全国文明城市、国家卫生城市、国家森林城市、全国双拥模范城等多项桂冠,极大地提升了城市美誉度和竞争力。成功争取并落地了一系列重大项目。27个项目纳入省级重点支持计划盘子,城建、能源交通、水利、社会事业、环境保护等101个省级重点项目落地建设。兰渝铁路广安支线全面建成,开通广安至成都动车、至重庆快车,巴广渝、遂广高速广安段建成通车,广安市过境高速公路东环线及渝广高速支线实现开工,广安港投入运营,回龙寺水库、龙滩水库加快建设,远成智慧物流、装配式建筑制造等重大项目开工落地,为全市经济社会发展积蓄了力量。成功争取并举办了一系列重大活动。成功举办邓小平同志诞辰110周年纪念活动,邓小平故里创建为国家5A级旅游景区;广泛开展“感恩作贡献”活动,积极争取社会各界支持,掀起了缅怀小平、感恩奋进的热潮。成功举办(承办)中国旅游日主会场启动仪式、全国残运会和特奥会圣火采集、国际红色马拉松赛、中国(广安)川渝合作示范区暨川东北经济区投资贸易大会、中国华蓥山旅游文化节、重庆经济协作区第十八次市长联席会等重大活动,广安知名度和对外影响力大幅提升。

五年来,我们坚持新常态要有新作为,综合施策稳定经济增长,全市经济综合实力迈上新台阶。积极应对经济下行不利影响,制定出台一系列稳增长政策措施,从多方面稳定了经济增势,经济实现了稳中有进、稳中向好发展。全市地区生产总值年均增长10.7%、突破1000亿元;人均地区生产总值是2011年的1.6倍。地方一般公共预算收入由27.8亿元增加到63亿元,年均增长17.8%。充分发挥投资的关键作用。主动对接国家重要投资方向,突出抓好基础设施、产业发展、民生改善等领域投资,固定资产投资年均增长25%,总量达到4543.7亿元;民间投资快速增长,年均增长24.7%、占比达到54%。充分发挥消费的基础作用。积极实施市场拓展“三大行动”,建成一批商务集聚区、大型卖场和专业市场,城乡商贸设施日臻完善。信息消费、旅游休闲、健康养老等消费不断扩大,社会消费品零售总额年均增长13.9%、达到2011年的2.1倍,成为拉动经济增长的重要引擎。充分发挥外贸的助推作用。广安获批并建成营运川东北地区首个公用型保税仓,新培育外贸进出口企业57户,外贸出口总额居全省前列。累计利用外资3.4亿美元。充分发挥实体经济的主体作用。建立支持实体经济发展、工业应急转贷、企业贷款风险补偿等专项资金,为企业提供各类资金支持7.1亿元;依托资本市场拓宽融资渠道,培育本地上市企业1家、“新三板”挂牌企业2家、“四板”挂牌企业46家。积极争取天然气直供、直购电试点及富余电量消纳政策,帮助企业降低生产成本1.15亿元,规上工业企业利润总额年均增长1.9%。

五年来,我们着力推动创新转型发展,加速培育发展新动能,经济实现更有质量更有效益增长。大力实施创新驱动发展战略,坚定不移转方式调结构,三次产业比重由19:51.4:29.6调整为15.8:51.2:33,高新技术产业总产值占规上工业比重提高8个百分点,科技对经济的贡献率提升到49.5%。民营经济实现增加值2631.4亿元,占GDP的比重达57.4%。“工业强市”战略深入实施。全面实施“中国制造2025广安行动计划”,成立重大产业发展推进工作组,加快推进电子信息、生物医药、精细化工、新型住宅制造、高端装备制造等八大重点产业发展,产业产值占工业总产值比重提高至62.8%;工业总产值、增加值年均分别增长15.3%、11.9%。推动电力、建材等传统产业技改扩能、转型升级,累计实施工业技改项目千余个,完成技改投资662亿元。大力化解过剩和落后产能,关闭退出地方小煤矿53户,整合水泥企业2户,淘汰落后产能117万吨。大力实施大企业大集团培育计划,规上工业企业较2011年新增124户,新培育小微企业5950户。坚持“一园一主业”,持续推进成熟园区建设,园区产值年均增长18.9%,广安经开区、邻水经开区分别纳入全省1000亿元和500亿元重点产业园区。现代服务业发展提质增效。加快实施省级现代服务业综合改革试点市、省级现代物流试点示范市和省级电子商务示范市建设,重点推进六大新兴先导型服务业发展,扎实推进服务业集聚区和重点项目建设,服务业增加值年均增长10.8%,占GDP的比重提升3.4个百分点。深入实施“互联网+”行动计划,积极推进电商物流与实体经济融合发展,电商交易额突破100亿元。旅游总收入年均增长22%以上。现代农业快速发展。建成"111"产村相融示范区650平方公里、现代农业产业基地205万亩,粮食总产实现十连增,农民人均可支配收入增速连续八年高于全国全省。新培育新型农业经营主体6624个,带动农户面达64.9%。成功创建邓小平故里优质农产品“华蓥山”公用品牌,广安农产品的市场占有率不断提升,产村相融、农旅结合、绿色发展的现代农业发展经验在全国全省推广。创新创业活力充分释放。加强科技创新平台建设,建立清华大学广安新能源研究院、赛迪工业和信息化研究院广安分院等7个重点产业技术研究院,新培育国家级高新技术企业33家,实施科技成果转化项目124个。我市整体创建为四川省可持续发展试验区、国家科技创新工程四川省试点城市,国家知识产权试点城市创建取得积极进展。大力推动“大众创业、万众创新”,建成省级创业示范基地(孵化园)3个。加快实施质量强市战略,“广安造”产品竞争力进一步增强。

五年来,我们创新实施多点多极支撑发展战略,坚持城乡统筹发展,城乡一体化快速推进。调整行政区划设立前锋区,扩大国家经开区管辖范围,设立新的市级园区,推动国有资产重组和管理体制改革,形成了多点驱动、竞相发展新格局。新型城镇化加快推进。广安市城市总体规划(2013-2030)获省政府批复,成功争取中国新型城镇化建设示范市、省级海绵城市建设试点城市;城市规划区协调发展,新拓展城区面积56.3平方公里、城镇化率年均提高1.75个百分点。大力实施“百镇建设试点行动”,在全市启动建设50个特色集镇,协兴等13个乡镇被列为全国重点镇,恒升等14个乡镇被列为全省百镇建设试点镇;建设幸福美丽新村662个,惠及群众121万人。城乡基础设施大幅改善。全市新增高速公路通车里程140公里;广安至岳池城市快速通道建成通车;广安至华蓥城市快速通道即将建成;开通广安主城区至岳池、前锋城市公交;广安客运枢纽站投入使用;新改建农村公路5000公里,实现100%乡镇通油路、100%行政村通公路。扎实推进农田水利建设,新开工建设中型水库5座,建成高标准农建示范区41.75万亩,整治农村集体土地建成高标准农田112万亩,治理水土流失219平方公里,解决112.7万农村居民和学生饮水安全问题,农建综合管理夺得全省“李冰杯”一等奖。大力实施“宽带中国”战略,4G网络城区、农村覆盖率分别超过97%、70%。县域园区发展活力迸发。夯实县域经济底部基础,做实园区发展载体平台,制定出台支持县域经济改革发展21条政策措施,6个区市县经济年均增速超过10%,广安经开区、枣山园区、协兴园区经济总量均突破50亿元,市属国有企业实力不断增强,资产总规模达204.63亿元。

五年来,我们全面深化改革充分开放合作,不断增创发展新优势,发展动力活力加速释放。重点领域改革全面推进。制定出台4个专项改革方案,1项改革纳入国家改革试点。“五证合一、一照一码”全面实施,累计新增各类市场主体28957个。全面完成农村土地确权登记颁证工作,农村土地流转率达34.4%;六区市县全域纳入全省首批农村产权抵押融资试点县。深化金融领域改革,成功引进成都银行、重庆银行、成都农商行、中银富登村镇银行,大力推进信用联社改制,全市新增银行业金融机构6家、保险机构9家、证券机构4家。推广运用政府和社会资本合作模式,启动实施PPP项目8个、总投资166亿元。供给侧结构性改革有序推进。公共资源交易、国资国企、公务用车、不动产统一登记制度改革成效显著。开放合作水平大幅提升。不断深化与重庆、深圳、天津、中关村等区域务实合作,邻水机电、高滩空港、华蓥电子信息等渝广合作配套产业园快速发展,15平方公里广深合作产业园启动建设,设立广安高新区作为中关村科技创新产业基地,与天津滨海新区建立战略合作关系,规划建设天津高新区(广安)创新示范园。深化拓展与韩国龟尾市的国际友好城市关系,积极参与四川与俄罗斯伏尔加河沿岸联邦区域合作,对德合作取得积极进展。投资促进工作成效显著。全市签约项目3643个、累计完成投资3093.43亿元。

五年来,我们务实推进绿色低碳循环发展,加快建设生态文明新家园,美丽广安建设迈出坚实步伐。生态建设深入推进。实施主体功能区规划,开展生态控制线划定工作,划定林业生态红线。完成营造林任务203.85万亩,森林覆盖率达到40.5%。荣获中国绿色城市奖、2014年度中国最佳绿色生态城市。大熊猫野化放归培训基地落户华蓥山。环境治理扎实有效。全面开展“洁净水”行动,嘉陵江、渠江出境断面水质达标,城市、乡镇集中式饮用水源地水质达标率分别为100%、93.7%,最严格水资源管理制度全面实施。切实加强大气污染联防联治,城市空气质量持续改善。科学治理土壤污染、农村面源污染,全市土壤环境质量总体良好。严格落实耕地保护制度,扎实开展永久基本农田划定,圆满完成省上下达耕地保护、基本农田保有量目标。实施完成28处重大地质灾害和15处矿山环境恢复治理,避险搬迁安置农户4700余户。节能减排成效明显。GDP、工业增加值能耗累计分别下降28.21%、61.75%,全面完成省政府下达的节能减排任务。广安经开区列为全国循环化改造示范试点园区,武胜县、广华工业园等纳入全省“十二五”首批循环经济试点单位。

五年来,我们紧紧扭住脱贫攻坚和民生改善重点,加快补齐全面小康短板,人民群众的获得感幸福感不断增强。每年坚持办好一批民生大事实事,财政民生投入占当年公共财政支出稳定在65%以上,实施省、市民生工程660项、办理惠民实事20余万件。城乡居民收入年均分别增长10.7%、12.2%,城乡收入比由2.6:1缩小为2.2:1,被评为全国优秀民生改善典范城市。脱贫攻坚卓有成效。累计减少贫困人口20.1万人,贫困发生率由8.9%下降到3.27%。保障体系更加健全。累计新增城镇就业22.31万人,城镇登记失业率持续控制在4%以内。养老、医疗、工伤、生育、失业等各类保险事业全面发展,全市基本养老保险覆盖人数达192.06万人,城镇基本医疗保险参保人数达89.62万人。教育事业健康发展。部省共建广安教育改革发展试验区建设取得丰硕成果,学前教育毛入学率提高14个百分点,华蓥市、武胜县、邻水县通过国家义务教育均衡发展验收,广安区、前锋区通过省级验收,普通高中高考本科上线近6万人,中职教育招生和在校生人数双增长,广安职业技术学院创建为省级示范性高职院校。吸引民间资金20余亿元投入教育事业,民办学校在校生人数达学生总数的23.4%。体育事业快速发展,竞技体育成绩斐然,全民健身活动蓬勃开展。卫生事业加快发展。健康广安建设扎实推进,基层医疗卫生标准化水平大幅提升,市县两级公立医院全部取消药品加成,成功争取为国家城市公立医院综合改革试点市,新增民营医疗机构31家,全面深化与华西医院、华西第二医院的医疗合作。120急救体系覆盖城乡,传染病发病率、孕产妇、婴儿死亡率连续多年低于全省平均水平。全面实施两孩政策,人口自然增长率、符合政策生育率控制在省定目标。文化事业持续繁荣。广安大剧院等文化设施建成开放,市博物馆开工建设,覆盖城乡的公共文化服务体系建设全面推进。群众文化活动多姿多彩。文艺创作成果丰硕,20余部文艺精品荣获中省奖项。文遗保护成效显著,全国重点文物保护单位增至5处,省级非遗项目达到9个。文化产业加速发展,产值较2011年提升355%。

五年来,我们全面落实依法治市方略,加强和创新社会治理,法治广安平安广安建设取得新成效。深入实施依法治市纲要,扎实开展“法律七进”活动,“六五”普法经验全省推广。深入推进依法治安,扎实开展危化品、建筑施工、消防、道路交通、煤矿和非煤矿山等领域安全专项整治,连续10年未发生重特大事故,安全生产形势持续稳定。加强和创新市场监管,深入开展假冒伪劣商品整治等专项行动,市场经营秩序持续好转。圆满完成食品药品监管体制改革,食品药品安全保障水平不断提升。切实加强农产品质量安全监管,成功创建四川省农产品质量安全监管示范市。着力维护社会稳定,严厉打击刑事犯罪和经济犯罪,全面开展禁毒行动,深入推进“五大民生警务”和“五小问题”解决,“互联网+公安”综合服务平台上线运行,网格化服务管理经验全省推广,立体化社会治安防控体系不断健全。持续开展信访“三无”创建活动,广安连续五年被人民网表彰为网民留言办理先进单位。全面推进社会稳定风险评估全覆盖,深化矛盾纠纷多元化解机制建设。依法治理民间非法投融资,社会大局持续稳定。

五年来,我们切实加强政府自身建设,推进政府服务管理创新,法治政府、廉洁政府和服务型政府建设取得新进展。完善政府决策程序规定,建立市政府常务会会前学法制度和法律顾问制度,加强规范性文件制定,启动政府立法工作,制定重要规范性文件76件。持续深化“放管服”改革,建立“三张清单”制度,市本级取消170项行政审批事项,全面取消非行政许可审批事项,建立行政审批中介服务事项目录清单;建成行政权力依法规范公开运行平台和电子政务大厅,实现省市县三级互联互通。建成行政执法和刑事司法衔接信息共享平台。瞄准权力运行重点领域和关键环节,在政府投资项目管理、土地出让、政府采购、PPP项目建设、招投标等领域制定出台一系列制度规定,积极推行“双随机、一公开”监管改革,切实把权力关进制度“笼子”。主动接受人大、政协监督,共办理市人大代表建议756件、政协提案1288件。深化政府信息公开,大力推行“网络理政”“在线访谈”,开通"12345"政府服务热线,有效促进了政民互动。落实全面从严治党、从严治政及党风廉政建设规定,扎实开展党的群众路线教育实践活动、“三严三实”专题教育和“两学一做”学习教育,政风行风持续转变。强化财政、审计监督和行政监察,支持执纪执法机关查办案件,旗帜鲜明反对腐败,政府自身建设不断加强,树立了为民务实清廉的良好形象。

同时,国防动员和后备力量建设、双拥共建、人民防空工作得到加强,统计服务、国资管理、粮食储备、食盐储备、应急管理、防震减灾、住房公积金管理、信息化建设、供销、气象、外事、侨务、港澳台事务、民族宗教、档案、保密、地方志、老龄事业、邮政、烟草专卖等工作取得新成绩,石油、电力、供水、通信等保障工作取得新进展,工会、共青团、妇联、科协、残联、贸促会、社科联、文联、红十字会、关工委以及各民主党派、工商联等作出新贡献。

各位代表,刚刚过去的2016年,面对宏观经济下行压力传导的深刻影响,我们积极应对、主动作为,全力推进改革发展稳定各项工作,全市经济进中提质、民生持续改善、社会和谐稳定、政风持续好转,实现了“十三五”良好开局。一是经济发展稳中向好。全市预计完成(下同)地区生产总值1060亿元、增长7.8%,固定资产投资1296亿元、增长14.5%,规上工业增加值增长8.4%,社会消费品零售总额467亿元、增长13%,一般公共预算收入63亿元、增长15.5%,一般公共预算支出234.8亿元、增长5.5%,全市银行业金融机构存贷款余额分别增长16%、12%。二是重点产业加速培育。新兴产业快速成长,生物医药、电子信息、高端装备制造、新型住宅制造等八个重点产业实现产值958亿元、同比增长26.6%,拉动工业增长14.9个百分点。现代服务业蓬勃发展,新产业、新模式、新业态加速培育,实现增加值350亿元、增长8.5%。现代农业提质扩面,“一路两特”产业基地加快建设,巩固提升现代农业产业基地13.8万亩。三是项目投资支撑有力。“项目年”工作扎实有效,666个省市县重点项目完成投资900亿元、增长20%以上;争取中央预算内补助资金支持项目132个、到位资金12.2亿元,申报获批国家专项建设基金项目3批86个、安排资金50.9亿元。招商引资项目到位资金达520亿元、增长3.3%。四是创新改革扎实推进。创新创业充满活力,新登记私营企业3086户、个体工商户16666户,同比分别增长26.9%、17.78%。新申报国家级高新技术企业9家,实施专利转化项目109项,实现专利转化产值98亿元,新培育中国驰名商标1件、著名商标6件、国家地理标志1件。供给侧结构性改革成效明显,化解房地产库存80万平方米,淘汰落后产能2万吨,协调金融机构为小微企业发放贷款123.4亿元。全面推进“营改增”改革试点,实现减税7996万元。五是城乡区域加快发展。成功申报省级海绵城市建设试点城市,50个特色集镇加快建设,城乡市政和公共服务设施完成投资57.8亿元。黑化农业产业大道156公里,新改建中小型水库42座、渠道178公里。县域经济实力不断壮大,园区发展活力不断增强,底部基础更加夯实。六是脱贫攻坚首战告捷。全市145个村退出贫困村,减少贫困人口50001人,贫困发生率下降到3.27%,广安区、前锋区、华蓥市即将“摘帽”。七是民生事业协调发展。民生支出达165亿元,占地方一般公共预算支出比重达70.3%。全面或超额完成省市民生工程任务。全市城镇新增就业5.51万人,失业人员再就业1.27万人,城镇登记失业率控制在3.68%。城乡居民收入分别达28158元、12554元,分别增长8%、10.4%。

各位代表,回顾发展历程,我们倍感欣慰。过去的五年,是广安经济实力显著增强、发展质量效益明显提升的五年,是全面深化改革攻坚破难、开放合作取得重大突破的五年,是脱贫攻坚卓有成效、民生社会事业加快发展的五年,是依法治市开启新征程、民主法制建设取得新进展的五年。五年的发展实践,使我们深刻地体会到:必须始终保持专注发展转型发展战略定力,主动适应引领经济发展新常态。始终坚持新常态要有新作为,主动应对“三期叠加”的不利影响,在加快发展中转型升级,在转型升级中追赶跨越,推动经济实现更有质量更有效益更可持续增长。必须始终用好改革开放、创新驱动强劲引擎,推动广安走在全省改革开放创新发展前列。坚定不移地走改革开放和创新发展之路,着力推动对内改革、对外开放,深入实施创新驱动战略,不断厚植发展新优势,增强发展新动能,开创发展新局面。必须始终树立以人民为中心的发展思想,努力让发展成果更多惠及人民群众。始终坚持以人为本、执政为民,把保障和改善民生作为政府最大责任,下功夫解决一批事关群众切身利益的民生难题,不断增进民生福祉。必须始终坚持依法行政、规范用权执政理念,着力构建遵规守纪的法治良序。坚持把依法行政贯穿政府工作各个环节,把权力关进制度的“笼子”,用法治的思维和方式推进政府工作,让遵规守纪成为自觉成为习惯,以法治、清廉、务实、高效的政府形象服务于民、取信于民、回报于民。必须始终坚持党的领导,全面加强政府系统党的建设。牢固树立政治意识、大局意识、核心意识和看齐意识,认真落实中央、省委和市委各项决策部署,做到党建工作与经济工作同谋划、同部署、同考核,把全面从严治党要求体现到政府工作方方面面,为推动广安跨越发展提供坚强保障。

各位代表,过去五年我市改革发展成就来之不易,这是省委省政府和市委坚强领导的结果,是全市人民克难攻坚砥砺奋进的结果。在此,我代表市人民政府向全市干部群众,向人大代表、政协委员,向各民主党派、工商联、无党派人士、人民团体和各界人士,向驻广部队、武警官兵和公安民警,向所有支持广安改革发展的同志们、朋友们,表示衷心的感谢!

我们也清醒地认识到,我市经济社会发展中仍然存在不少困难和问题:经济下行压力传导影响加深,产能过剩和需求结构升级矛盾突出,经济增长内生动力不足;区域发展不平衡、城乡发展不协调问题仍然突出,民生社会事业还存在薄弱环节,全面建成小康社会还需补齐短板;协调各方利益、维护社会稳定压力加大,公共安全隐患不容忽视,环境治理、食品安全、安全生产形势依然严峻;政府职能转变还需深化,作风建设任重道远,依法行政和反腐倡廉仍需持续用力。对此,我们将以对党和人民高度负责的态度,认真解决这些问题,决不辜负全市人民的期望!

今后五年工作建议

今后五年,是我市决胜全面小康、建设经济强市的攻坚阶段。我们面临的最大矛盾是发展不足,最大难题是转型跨越,最大机遇是创新改革,最大责任是富民强市。综观国内外形势,宏观环境日益复杂,转型压力日益增大,区域竞争日趋激烈,社会诉求日益多元,不可预见性制约不断增多。但审视发展现状,有利因素多于不利因素,发展机遇大于困难挑战,广安仍处于大有作为的重要战略机遇期。发展基础更加坚实。全市地区生产总值和全社会固定资产投资均突破千亿元,战略性新兴产业发展态势良好,一批重大产业项目陆续投产,新的增长动能正加快形成;工业化城镇化迈入加速期,现代综合交通体系逐步完善,广安区、前锋区、华蓥市即将脱贫摘帽,决胜全面小康、建设经济强市具备了坚实基础。发展机遇更加凸显。国家“一带一路”、长江经济带、西部大开发、成渝城市群、四川全面创新改革试验区、四川自贸区等战略深入实施,广安在国家中小城市综合改革试点城市、国家循环经济示范城市、国家现代农业示范区等方面享有先行先试的支持政策,为转型发展科学发展创造了良好条件。发展空间更加广阔。川渝合作示范区、国家西部承接产业转移示范区等国家战略持续发挥效应,广安与重庆、深圳、天津、中关村等重点区域深度合作,正成为四川对外经济走廊的重要节点,不断释放出巨大的发展活力。只要我们坚持不懈、奋发有为,在新一轮发展中抢占先机、争抓主动,努力把困难和挑战化为动力,把机遇和潜力变为现实,就一定能实现发展新跃升、转型新跨越,如期与全国全省同步全面建成小康社会。

今后五年政府工作的总体要求是:坚持以中国特色社会主义理论体系为指导,深入学习习近平总书记系列重要讲话精神,紧紧围绕统筹推进“五位一体”总体布局和协调推进“四个全面”战略布局,牢固树立和贯彻落实新发展理念,全面落实市第五次党代会精神,坚持新常态有新作为,始终保持专注发展转型发展定力,深入实施“三大发展战略”,统筹做好稳增长、促改革、调结构、惠民生、防风险各项工作,以全面创新改革引领经济转型发展,着力推进供给侧结构性改革,加快培育发展新动能,促进发展成果共享,奋力推进广安向全面小康、经济强市跨越。

根据市第五次党代会提出的奋斗目标,建议在全面完成“十三五”规划、全面建成小康社会的基础上,今后五年广安经济社会发展的主要目标是:发展速度保持快于全国全省。经济发展方式转变取得决定性进展,全要素生产率明显提高。改革创新实现重大突破,发展新动能加速成长,圆满完成国家中小城市综合改革等试点任务。开放合作取得显著成效,开放型经济水平大幅提升,全面建成川渝合作示范区和国家西部承接产业转移示范区。绿色发展理念深入人心,资源节约型和环境友好型社会建设取得重大进展,全面实现国家循环经济示范城市和国家生态文明先行示范区建设目标。社会事业全面进步,人民生活水平和质量普遍提高,城乡居民人均收入达到全国全省平均水平,与全国全省同步全面建成小康社会。

按照上述目标要求,必须以新发展理念为指导,重点抓好以下几个方面。

一是坚持把发展基点放在创新上,以创新驱动引领转型发展。坚定走创新驱动转型发展之路,坚决打好格局优化、动力转换、结构调整硬仗。创新培育现代产业体系。加快发展电子信息、生物医药、精细化工、智能制造、新材料、新能源等战略性新兴产业,加快改造电力、建材等传统产业,打造新型工业发展高地。加快发展电子商务、现代物流、现代金融等新兴先导型服务业,积极培育新业态新模式,巩固提升以邓小平故里、华蓥山旅游文化景区为核心的旅游发展格局,推动服务业智能化、低碳化、绿色化发展。全面推行优质农产品标准化、规模化、品牌化生产,推动农业与旅游、康养等产业深度融合,提升国家现代农业示范区建设水平。全面加强创新能力建设。抓住新科技革命、产业变革、数字经济等历史性机遇,推进产业技术创新、区域协同创新、大众创业万众创新,打造创新基地、众创空间和科技孵化器等创新平台,培育高新技术企业。借助高端决策咨询暨投资促进专家等优势,积极推进新技术、新专利、新成果转化为高新技术产业。加快构建创新发展体制机制。持续推进全面创新改革试验,健全以企业为主体的技术创新机制、促进科技成果应用转化机制、金融对接服务科技创新机制、创新人才培养引进和激励机制等,推动与中关村建立协同创新共同体,充分激发全社会创新活力。

二是坚持对内改革对外开放,持续增强发展内生动力。深入实施改革开放战略,加快形成更加全面、更加深入、更加多元的改革开放新格局。纵深推进重点领域改革。按照中央和省委、市委改革既定部署,系统谋划出台一批重大改革方案,加快形成落实“五位一体”总体布局、“四个全面”战略布局的制度机制。以供给侧结构性改革为主线,深化“放管服”、农业农村、财税金融、投融资、公共资源交易等重点领域改革,推动各项改革取得突破性进展。务实推动对外开放合作。精准对接国家“一带一路”、长江经济带、成渝城市群等发展战略,加强与德国、日韩、港澳台等国家和地区合作,全面深化与长三角、珠三角、环渤海,以及重庆、深圳、天津、中关村等重点区域合作,打造内陆开放型经济高地。持续办好中国(广安)川渝合作示范区暨川东北经济区投资贸易大会、中国华蓥山旅游文化节等重大节会,推动招商引资取得更大实效。大力发展外向型经济。优化对外贸易和外向型经济结构,支持和发展生产型出口企业,确保进出口总额走在全省前列。借助四川自贸区、重庆自贸区等平台,加快检验检疫、海关监管区便利化建设,全面提升外向型经济水平。

三是坚持城乡统筹协调发展,加快推进新型城镇化进程。尊重和顺应城镇化规律,促进城乡统筹一体化发展。提档升级中心城区。改造升级老城区,配套完善公共服务设施,打造现代服务业集聚区和宜居宜业综合居住区。加快奎阁、官盛、神龙山组团等片区建设,打造高新技术产业集聚区和生态型综合居住区。推进省级海绵城市试点市建设,加快建设地下综合管廊、城市停车场等基础设施,不断提升主城区综合承载能力。加快建设组团城市。按照“一主、四副、轴带引领”网络化城镇空间布局,快速推进县城、中心集镇建设,加快建设主城区至区市县快速通道,开通组团城市公交,促进中心城区与组团城市融合发展。配套完善城乡功能。加快完善铁路、高速公路网络,升级改造国省道和农村公路,完成渠江航道整治,争取建设广安支线机场、通用航空机场。加快建设一批重大水利工程,改造升级农村电网、4G网络,切实改善群众生产生活条件。统筹建设美丽乡村。深入开展“百镇建设试点行动”,重点打造一批特色集镇,示范带动城乡协调发展。坚持产村相融、农旅结合,加快建设幸福美丽新村。

四是坚持绿色发展生态优先,着力构建生态文明新格局。牢固树立绿色发展理念,坚持走生产发展、生活富裕、生态良好的文明发展之路,加快推进美丽广安建设。大力发展绿色低碳循环经济。以创建国家循环经济示范城市为契机,全面推进传统制造业绿色改造,积极发展节能环保和清洁能源产业,推进智能制造、绿色制造,加快发展现代服务业,大力发展有机农业、生态农业。加快推进生态主体功能区建设。按照人口、资源、环境相均衡的原则,统筹衔接各类空间规划,合理开发利用资源。严守资源消耗上限、环境质量底线、生态保护红线。加大环境污染防治力度,全面推行河长制,深入实施“洁净水”行动,集中打好水、大气、土壤污染防治攻坚战,全面提升生态环境质量。积极构筑长江上游生态屏障。以嘉陵江流域国家生态文明先行示范区建设为抓手,推进大规模绿化广安行动。推进重点区域水土流失、石漠化综合治理,加强湿地保护,进一步增强生态系统稳定性和生态服务功能。健全防汛减灾及地质灾害监测预警体系、防治体系、应急体系,全面提升防汛及地质灾害防治水平。

五是坚持发展成果人民共享,不断增进民生福祉。主动顺应人民群众新期待,加快补齐全面小康短板,让改革发展成果更多惠及人民群众。进一步提升民生保障水平。持续加大民生投入,办好民生工程和民生实事。健全社会保障体系,加快发展残疾人、慈善、福利、优抚、救助等事业,筑牢全面小康兜底保障线。坚决打赢脱贫攻坚战,确保全市所有建档立卡贫困户全部脱贫、820个贫困村全部退出、6个贫困区市县全部摘帽。进一步增强公共服务能力。优先发展教育事业,巩固提升部省共建教育改革发展试验区建设成果,推进广安职业技术学院创建应用型本科院校。推进健康广安建设,深化公立医院改革,确保人群主要健康指标达到或高于全国水平。推动群众体育、竞技体育、体育产业协调发展。扎实推进妇女儿童事业发展。进一步推动文化事业发展。巩固提升全国文明城市成果,大力弘扬社会主义核心价值观,培育社会文明风尚。推动文化与旅游融合发展,文化事业与城市建设互动发展,不断提升广安软实力。进一步推进社会治理创新。健全系统治理、依法治理、综合治理、源头治理工作机制,正确处理好维护秩序与激发活力、打击犯罪与维护权益、严格执法与热情服务的关系,深化平安广安建设,健全公共安全体系,坚决守住社会稳定、安全生产、食品药品安全“三条底线”,切实提升人民群众安全感。

2017年工作重点

2017年是实施“十三五”规划承前启后的重要一年,也是新一届政府各项工作的开局之年。我们要按照中央、省委经济工作会议和省委省政府、市委的部署,坚持稳中求进工作总基调,牢固树立和贯彻落实新发展理念,适应把握引领经济发展新常态,以提高发展质量和效益为中心,以推进供给侧结构性改革为主线,深化创新驱动,推进改革开放,促进经济社会平稳健康发展,以优异成绩迎接党的十九大和省第十一次党代会胜利召开。

综合各方面因素,建议全市经济社会发展的主要预期目标是:地区生产总值增长8%左右,全社会固定资产投资增长12%,社会消费品零售总额增长11%,地方一般公共预算收入增长10%,城乡居民人均可支配收入分别增长8%、9%;完成省上下达的节能减排任务。重点做好以下工作:

一、持续推动经济稳定增长

努力扩大有效投资。继续开展“项目年”活动,重点推进广安过境高速公路东环线及渝广高速支线等214个重大基础设施项目,杭萧钢构等210个重大产业项目,市博物馆等140个重大民生项目,固定资产投资完成1450亿元以上。主动对接国家重大投资工程包、重点专项规划、三年投资滚动计划和专项建设基金,策划实施重点项目600个以上,力争远成智慧物流等30个项目列入省级重点建设项目,广深产业示范基地一期等5个项目列入全省100个重点推进项目。加大工业和技改投资力度,强化项目跟踪服务,增强企业投资信心,完成工业投资280亿元以上、技改投资110亿元以上。充分激发民间投资活力,推动民间投资持续稳定增长。

持续推动消费转型。贯彻落实中省扩大消费政策措施,提质扩容幸福产业服务消费,围绕旅游、文化、体育、养老、教育培训等重点领域,提升服务品质,增加服务供给,不断释放潜在消费需求;推进商贸综合体、核心商圈、骨干专业市场建设,推动实体零售创新转型、线上线下互动,打造一批名店、名标、名牌,加快社区便民服务中心建设,完善社区商业网点布局,构建便民生活服务圈。全年实现社会消费品零售总额510亿元以上。

提质发展外经外贸。认真落实国家促进外贸稳定增长和提质增效系列政策,实施“外贸+”战略,推进优进优出“三大工程”,调优外贸产业产品结构,鼓励“广字号”企业开拓“一带一路”沿线市场,推动外经外贸企稳回升。积极争取海关、检验检疫设立机构,依托公用型保税仓开展平行进口汽车等业务,提升外经外贸发展水平。推进“自主品牌出口增长”计划,发展壮大华蓥省级电子信息出口基地、邻水省级汽摩产品出口基地,支持前锋创建省级轻纺专业出口基地。培育生产型出口企业15户以上。

二、着力推进产业转型发展

推动新型工业向中高端迈进。认真落实“中国制造2025四川行动计划”,发扬“工匠精神”,深入开展质量对标提升行动,扩大高质量产品和服务供给,规上工业增加值增长8.5%以上。加快发展电子信息、生物医药、新材料、新能源、高端装备制造等战略性新兴产业,力争产值增长15%以上。实施工业强基、制造业创新中心建设等工程,推进智能制造、绿色制造、工业云及工业大数据创新应用。加快利用新技术新业态,培育发展玄武岩纤维等潜力产业,改造提升电力、建材等传统产业,进一步提升产业发展层次和水平。完善检验检测、技术研发、物流运输、商务金融等专业配套,打造成熟园区,提升园区承载能力。全面实施企业培育工程,新培育规上工业企业35户以上。优化产业规划、市场开拓、要素保障、政策支持等企业帮扶机制,积极支持企业发展,推动工业经济稳定增长。

推动现代服务业向品质化跃升。优先发展电子商务、现代物流、现代金融、健康养老等新兴先导型服务业,加快发展咨询、研发设计、检验检测等生产性服务业,推动居民生活服务业转型升级,服务业增加值增长8.3%以上。加快省级电子商务示范市建设,建成广安区、前锋区、华蓥市电商集聚区,电商交易额增长20%以上。加快推进枣山、前锋等物流园区建设,开业营运远成智慧物流项目,物流业增加值增长10%以上。深入实施省“三百工程”和市“三五工程”,新培育限上商贸企业20户以上。启动广安国际会展中心建设,加快构建区域性会展中心城市。启动华蓥山国家生态旅游示范区建设,精心筹办第二届中国华蓥山旅游文化节、2017四川乡村文化旅游节、第四届四川国际旅游交易博览会主题市活动,旅游收入增长15%以上。

推动现代农业向价值链延伸。加快国家现代农业示范区建设,推进农业供给侧结构性改革,增加绿色优质农产品供给,提高农业综合效益和竞争力。优化农业产业结构,推进现代农业三产融合发展示范区建设,建成“一路两特”华蓥市禄市镇至前锋区大佛寺街道产业带,发展农产品深加工和电子商务、创意休闲农业等新业态,着力提高农业全产业链收益,努力做强一产、做优二产、做活三产。细化和落实承包土地“三权分置”办法,发展多种形式适度规模经营,培育新型农业经营主体和服务主体,促进农业提质增效和农民持续增收。狠抓农产品标准化生产、品牌创建、质量安全监管,持续营销“华蓥山”公用品牌。落实粮食安全行政首长责任制,抓好粮食安全生产。做好第三次农业普查。

三、系统推进全面改革创新

深化供给侧结构性改革。深入推进“三去一降一补”,推动五大任务取得实质性进展。稳妥化解过剩产能,抓住处置“僵尸企业”这个牛鼻子,运用市场、法治的办法引导煤炭、水泥等行业兼并重组、转型升级,压减煤炭产能39万吨、水泥产量400万吨。积极化解房地产库存,结合促进人口城镇化,大力发展住房租赁市场,积极推进棚改货币化安置,科学调控土地出让、房地产开发。有效防范金融风险,支持企业市场化、法治化债转股,加大股权融资力度,依法解决民间借贷和非法集资问题,防范和化解地方政府性债务风险。着力降低企业成本,认真落实中省减税降费政策,降低企业用能成本、物流成本,提高劳动力市场灵活性,推动企业眼睛向内降本增效。

推进一批重大改革事项。加快实施已出台的各项改革方案。加强“放管服”改革,深化商事制度改革,更大程度激发市场主体活力。深化农村集体资产股权量化改革,盘活农业农村资产。加强投融资体制改革,滚动推出一批PPP项目,形成区市县、园区PPP项目指导、储备、审批、实施的良性运行机制。加快金融体制改革,全面完成信用社改制,依托多层次资本市场,拓宽融资渠道。继续深化预算管理改革,加快转变财政支持产业发展方式,加大政府购买服务力度。有序推进环境保护督察、知识产权保护制度、公务用车管理、不动产统一登记制度、公共资源交易监管、国有林场、供销、社会保障等领域的改革。

释放全社会创新创业潜能。实施“创新广安”行动,认真落实支持创新创业政策措施,促进新动能发展壮大、传统动能焕发生机。加强创新平台建设,积极培育省级高新区和科技企业孵化器,建成广安科技企业孵化器服务平台。全面启动国家知识产权试点城市创建工作。深入实施商标品牌战略,新培育驰名和著名商标品牌6件。大力引进高层次创新型人才,加快形成区域人才高地。保护企业家精神,支持企业家专心创新创业。

四、务实推进全面开放合作

深度融入国家开放战略。主动参与“一带一路”建设,积极对接国际产能合作"111"工程,推动装备制造、电子信息、精细化工等项目纳入全省外经贸"251"三年行动计划重大项目库,鼓励优势企业开展国际产能、装备制造合作,深化与沿线国家资源开发、农业、旅游等合作。全方位融入长江经济带和成渝城市群建设,努力构建对外开放新格局。

全面深化重点区域合作。继续加强与长三角、珠三角、京津冀、成渝等区域合作,完成川渝合作示范区(广安片区)建设中期评估,主动融入成渝经济区和川东北经济区建设。加快推进广安(深圳)合作产业园、天津高新区(广安)创新示范产业园建设,力争在机制创新和项目落地上取得突破。

务实推进投资促进工作。优化完善投资促进机制,整合投资促进部门、驻外机构和重大产业推进工作组力量,促进区市县、园区招商信息共享、活动共办、项目共推。精心举办“感恩小平·投资广安”系列专题投资促进活动,积极参加渝洽会、农博会等重大投资促进活动,扎实抓好签约项目跟踪落地,切实提高招商项目履约率和资金到位率。全年力争签约引进项目570个、招商引资完成固定资产投资550亿元。

五、加快推进新型城镇化

突出抓好城市建设管理。科学制定和严格执行城市规划,完成渠江、西溪河两岸等重要节点规划设计,加快编制特色集镇、示范村庄规划。深化“城市基础设施建设行动”,推进省级海绵城市试点建设,持续改造城镇棚户区,推进奎阁、官盛、神龙山组团开发,加快地下综合管廊和城市停车场建设,完成充电桩、公共停车场规划布点,加快推进A号桥建设、滨江路综合改造、万盛片区雨污分流及道路改造,启动五福桥、广前大道城区段改造工程,增强城市综合承载能力。综合运用金融、土地、财税、投资等手段,促进房地产市场平稳健康发展。扎实推进以人为核心的新型城镇化,落实户籍制度改革方案和居住证制度,促进农民工市民化。推动规划管理地方立法和城市管理体制改革,完成城管大部门制改革。全市新增城镇面积8平方公里,城镇化率提高1.6个百分点。

加强城乡基础设施建设。加快广安过境高速公路东环线及渝广支线建设,推进枣山至港口至前锋快速通道及G212线、S203线、S204线、S208线、S406线等国省干线公路建设,力争开工建设广(安)镇(巴)高速公路。新改建农村公路1000公里以上。抓好亭子口灌区等重点水利项目前期工作,加快回龙寺、猫儿沟等重点水利项目建设,持续改善群众生产生活条件。改造升级农村电网,提高城乡供电保障。加快4G网络、宽带乡村建设,全面启动物联网基础建设,不断提升城乡信息化水平。

六、深入实施脱贫攻坚工程

聚焦“两不愁、三保障”和“四个好”目标,继续按照省委树旗帜、立标杆的要求,深入推进脱贫攻坚,实现266个贫困村退出、48164名贫困人口脱贫。

实施脱贫攻坚重点工程。抓好产业扶贫,强化利益联结机制,推动龙头带动、入股分红、资产收益等脱贫增收新模式,用好贫困村产业发展基金,完善产业扶贫收益分配政策,发展壮大村集体经济,稳定劳务输出,做到每个贫困家庭有1个增收产业或1人实现就业;全面巩固低保线和扶贫线“两线合一”,确保贫困群众不愁吃、不愁穿。抓好住房安全建设,统筹做好易地扶贫搬迁、新村建设、农村危旧房改造、地质灾害搬迁等工作,同步搞好路、水、电、通信、公共服务等基础设施配套,确保贫困群众住房安全有保障。抓好医疗教育扶贫,推动基层医疗教育机构软硬件达标,落实好医疗扶贫“十免四补助”等特殊支持政策,用好教育、卫生基金,解决好因病致贫返贫、因贫辍学等问题。抓好新风塑造,持续开展“四好村”创建活动,加强感恩奋进教育,引导群众自力更生、艰苦奋斗,养成好习惯、形成好风气。

健全脱贫攻坚推进机制。充分调动各方力量,落实“五个一”帮扶制度,动员社会各方面共同参与,形成推动脱贫攻坚的强大合力。深化精准扶贫精准脱贫,完善贫困户动态管理制度,切实做到因户因人施策。整合用好农业、交通、水利等涉农资金,加大财政扶贫资金投入,创新金融扶贫方式,引导社会资本参与扶贫,不断拓宽投入渠道。强化扶贫资金监管,提高扶贫资金使用效益。加强扶贫信息系统建设,适时监测脱贫对象、扶贫项目,深入实施脱贫成效第三方评估,确保真扶贫、真脱贫。

七、切实加强生态文明建设

扎实开展绿化广安行动。大力推进造林增绿,扩大新一轮退耕还林成果,完成乡村造林增绿12.9万亩、“一路两特”森林景观工程2.8万亩。打造渠江、嘉陵江等绿色生态廊道,完成江河湖库防护工程4.85万亩。实施交通廊道绿化,建设山体公园、街头绿化等城市生态景观。加强森林资源保护,推进白云湖国家湿地公园建设试点。

加大环境污染防治力度。全面贯彻“大气十条”“水十条”“土十条”等政策规定,加大水、大气、土壤污染防治力度,扎实开展“洁净水”行动,抓好乡镇污水处理厂(站)运行管理,确保城市集中式饮用水源地水质达标率100%,空气优良天数达标率77.9%以上。严格落实环境保护党政同责和一岗双责,持续推进环境保护网格化监管,强化环境安全隐患排查,抓好环境突出问题治理,加强环保基础设施建设运行监管,健全环保联动执法机制,强化生态环境损害责任追究,全面完成中省下达环境保护目标任务。

推动绿色低碳循环发展。深化国家循环经济示范城市创建活动,推进广安经开区国家循环经济示范试点园区改造,加快建设循环型企业、循环型园区。加快国家住宅产业现代化综合试点市、全省建筑产业现代化试点市建设,大力推广装配式建筑和绿色节能建筑。抓好工业、交通等重点领域节能降耗,加快淘汰落后产能和工艺设备。倡导绿色生活方式,促进绿色消费、绿色出行、绿色居住成为公众自觉行动。

八、统筹发展民生社会事业

办好一批民生实事。健全民生支出稳步增长机制,公共财政不低于65%投向民生领域。全面完成省定民生工程和民生大事。集中办好市定民生实事,实施扶贫帮困工程、基础设施工程、环境整治工程等8大类惠民项目,进一步织密民生保障网。

提升社会保障水平。实施更加积极的就业促进政策,城镇新增就业3.6万人,城镇登记失业率控制在4%以内。完善社会保障体系,持续深化“五险合一”征缴方式改革,扎实做好城乡居民医疗保险整合工作,稳步推进医疗保险异地就医即时结算。强化住房保障,开工改造城镇危旧房棚户区15915套以上、农房16500户以上。推进适度普惠的社会福利体系建设,新增(改造)养老床位3900张。完善临时救助制度,提高城乡低保、特困供养标准。规范慈善组织的登记和管理,大力发展慈善事业。

办好人民满意教育。深化部省共建广安市教育改革发展试验区建设,协调发展各类教育,切实提高办学质量水平。加快公办幼儿园和普惠性幼儿园建设,新建城市公办幼儿园7所。加快义务教育学校标准化建设,改善农村薄弱学校基本办学条件,切实解决城镇大班额,确保广安区、前锋区、岳池县顺利通过中省督导评估。促进普通高中多样化发展,推动中职教育内涵发展,加快普及高中阶段教育步伐,全面启动广安职业技术学院创建应用型本科院校。健全公共体育服务体系,大力发展体育产业,推动老年体育和全民健身活动健康发展,积极备战省第十三届运动会。

优化医疗卫生服务。建立健全四川大学华西医院与市、县、乡三级医疗机构的分级诊疗服务体系,加强与华西第二医院合作。加强基层医疗卫生机构标准化建设,深入推进公立医院综合改革,理顺医疗服务价格,完善医保支付方式,确保患病群众就近享受优质医疗服务。开展全民预防保健行动,积极防治重大传染病、地方病。

促进文化繁荣发展。大力实施文化惠民工程,健全完善覆盖城乡的公共文化服务体系。加快市文化传媒综合体、市博物馆等重点项目建设,立项建设广安市科技馆,加强重点文物保护,申报省级以上“非遗”项目4个。深入实施“文化产业倍增”计划,文化产业增加值突破20亿元。大力繁荣文艺创作,推出更多反映广安地方特色的文艺作品。

九、全面加强社会治理创新

创新社会综合治理。以村(社区)“两委”换届为契机,加快建设社区公共服务综合信息平台,增强社区服务功能,实现政府治理和社会调节、居民自治良性互动。持续推进网格化服务管理规范化建设,夯实基层社会治理基础。全面实施居民身份证异地换领制,推广使用农村交管平台,深化“五大民生警务”,优化“互联网+公安”综合服务平台,不断提升警务效能。加强政务诚信、商务诚信、社会诚信和司法公信建设,进一步健全信用体系。支持工会、共青团、妇联等群团组织参与社会治理,形成多元共治格局,不断提升社会治理水平。

深化平安广安建设。深入推进“七五”普法,不断提升社会法治化管理水平。健全落实社会治安综合治理领导责任制,持续开展严打整治专项斗争,营造安全稳定的社会环境。完善不稳定因素排查和矛盾纠纷多元化解机制,持续深化信访“三无”创建,加强“五小问题”解决,有效化解信访积案,严格依法规范信访秩序。健全落实重大决策、重大项目建设风险评估机制,加强社会稳定风险防范。

健全公共安全体系。深入落实安全生产“三个责任”,抓实重点行业领域安全生产,坚决遏制重特大事故发生,减少一般性事故。深入开展食品药品安全治理,加强应急管理,预防和减少突发事件发生,切实保障人民群众生命财产安全。做好全民国防教育和双拥工作,提升国防后备力量建设水平。

全面提高政府治理现代化水平

各位代表,站在新的历史起点,新形势新任务对政府工作提出了新的更高要求。我们将着力围绕推进治理体系和治理能力现代化,坚持以改革创新的精神加强政府自身建设,不断提高施政能力和服务水平,努力建设人民满意政府。

坚持依法行政、依法行权,切实规范行政权力。全面贯彻落实《法治政府建设实施纲要》和《依法治市纲要》,完善决策咨询和法律顾问制度,积极开展相对集中行政许可权改革试点,推进综合行政执法体制改革。依法接受人大及其常委会法律监督和工作监督,主动接受政协民主监督,重视司法、舆论和社会监督。

坚持务实勤政、崇尚实干,提升政府服务水平。持续深化“放管服”改革,继续清理行政审批事项,厘清政府与市场、社会的边界,进一步完善“三张清单”。建立政府购买公共服务的制度体系和管理平台,不断提高政府公共服务水平。探索适应大数据特征的政府服务管理新方式,加快推进“互联网+”政务服务,进一步提升行政效能。深化政务公开和政府信息公开,着力打造“阳光政府”。

坚持从严治政、正风肃纪,持续改善政风行风。落实全面从严治党、从严治政各项规定,认真落实新形势下党内政治生活若干准则和党内监督条例,切实加强政府系统党的建设。巩固深化“两学一做”学习教育成果,扎实开展三项整改“回头看”活动,推动作风建设常态化长效化。严格落实厉行节约反对浪费制度规定,严控“三公”经费支出,进一步提高财政资金使用效益。严格落实工作责任制、督查问责制、激励和容错纠错机制,坚决整治庸政懒政怠政行为,确保各项工作部署不折不扣落实。

坚持廉洁从政、惩治腐败,营造良好政治生态。认真落实党风廉政建设主体责任,加强反腐倡廉教育和廉政文化建设。坚持廉政建设与业务工作两手抓、两手硬,发挥行政监察和审计监督作用,突出重点领域、关键环节和重要岗位监督管理,坚决纠正和查处各种顶风违纪行为。始终保持惩治腐败高压态势,支持纪检监察机关严肃查处违法违纪案件;推进党风廉政建设向基层延伸,坚决查处侵害群众利益的不正之风和腐败问题,让人民群众切实感受清风正气。

各位代表,新起点谋划新蓝图,新征程更需新作为。让我们紧密团结在以习近平同志为核心的党中央周围,在省委、省政府和市委的坚强领导下,高举中国特色社会主义伟大旗帜,紧紧团结依靠全市人民,发扬“逢山开路、遇水架桥、披荆斩棘、勇往直前”的广安精神,开拓创新,务实奋进,推动广安加快迈入转型发展、科学发展快车道,为决胜全面小康、建设经济强市而努力奋斗!
